# The impact of antibiotic use in gastrointestinal tumors treated with immune checkpoint inhibitors: systematic review and meta-analysis

**DOI:** 10.3389/fmed.2024.1415093

**Published:** 2024-06-03

**Authors:** Faizah M. Alotaibi, Ibrahim Abdullah S. Albalawi, Amna M. Anis, Hawazin Alotaibi, Seham Khashwayn, Kanan Alshammari, Jaffar A. Al-Tawfiq

**Affiliations:** ^1^College of Science and Health Professions, King Saud Bin Abdulaziz University for Health Sciences, Alahsa, Saudi Arabia; ^2^King Abdullah International Medical Research Center, Alahsa, Saudi Arabia; ^3^Faculty of Medicine, Tabuk University, Tabuk, Saudi Arabia; ^4^College of Medicine, Alfaisal University, Riyadh, Saudi Arabia; ^5^Department of Biomedical Engineering, Imam Abdulrahman bin Faisal University, Dammam, Saudi Arabia; ^6^College of Medicine, King Saud bin Abdulaziz University for Health Sciences, Jeddah, Saudi Arabia; ^7^King Saud Bin Abdulaziz University for Health Sciences, Alhasa, Saudi Arabia; ^8^Department of Oncology, Ministry of National Guard Health Affairs, Riyadh, Saudi Arabia; ^9^King Saud Bin Abdulaziz University for Health Sciences, Riyadh, Saudi Arabia; ^10^King Abdullah International Medical Research Center, Ministry of National Guard-Health Affairs, Riyadh, Saudi Arabia; ^11^Department of Specialty Internal Medicine and Quality, Johns Hopkins Aramco Healthcare, Dhahran, Saudi Arabia; ^12^Infectious Disease Division, Department of Medicine, Johns Hopkins University School of Medicine, Baltimore, MD, United States; ^13^Infectious Disease Division, Department of Medicine, Indiana University School of Medicine, Indianapolis, IN, United States

**Keywords:** antibiotic, immune checkpoint inhibitor (ICI), anti-PD-1, gastrointestinal cancers, hepatocellular carcinoma (HCC), esophageal cancer, esophageal squamous cell carcinoma

## Abstract

**Background:**

Immune checkpoint inhibitors (ICI) have improved overall survival in patients with different cancer types. However, treatment efficacy varies between patients depending on several factors. Recent research suggested that antibiotic-induced dysbiosis can impair ICI efficacy. Here we review the impact of antibiotic use in clinical outcome of patients with gastrointestinal cancer treated with ICI.

**Methods:**

This is a systematic review and utilized a thorough search of MEDLINE, Cochrane, Scopus, EB-SCO, Web of Science of studies published till September 2023. The aim of the study is to determine the association between antibiotic use and ICI treatment efficacy in patients with gastrointestinal cancers (GI). We utilized a meta-analysis of the association between the use of antibiotics and overall survival and progression-free survival.

**Results:**

Nine studies met the inclusion criteria with a total of 2,214 patients. The most common type of cancers was hepatocellular carcinoma (HCC). The majority of the studies were retrospective, and one was collective of clinical trials. The use of antibiotics was associated with decreased both overall survival [haz-ard ratio (HR) 1.92, 95% confidence interval (CI) 1.41, 2.63] and progression-free survival [HR 1.81, 95% CI 1.29, 2.54].

**Conclusion:**

The use of antibiotics may affect clinical outcomes in patients with GI cancers treated with ICI. Further prospective studies are needed to improve the understanding of this phenomenon.

**Systematic review registration:**

https://www.crd.york.ac.uk/prospero/display_record.php?ID=CRD42023462172.

## 1 Introduction

Gastrointestinal (GI) cancers, mainly hepatocellular carcinoma (HCC), gastric cancer, pancreatic cancer and colorectal cancer (CRC), are of the common cancers worldwide (1). These cancers, derived from the GI system and related digestive organs, have different clinical features but may share similar characteristics. There are few treatment options for gastrointestinal malignancies, particularly for those that are hard to resect and have metastasized. In contrast to other conventional therapies, immunotherapy, such as immune checkpoint inhibitors (ICI), offers a promising method for treating a variety of cancers, particularly GI cancers, and can produce a long-lasting response (2). Currently approved-ICIs such as monoclonal antibodies (mAb) targeting programmed cell death protein 1 (anti-PD-1) (Nivolumab, Pembrolizumab, and Cemiplimab), anti-PD-1 ligand (anti-PD-L1) (Atezolimumab, Durvalumab and Avelumab), and cytotoxic T-lymphocyte-associated protein 4 (anti-CTLA-4) (Ipilimumab) have revolutionized cancer treatment and resulted in improved clinical outcomes for many types of cancers (3). However, a large number of GI cancer patients who received ICI treatment go on to develop primary or secondary resistance to the medication. In order to anticipate response to ICI and overcome resistance, it is necessary to comprehend the underlying mechanism, develop biomarkers and investigate new therapeutic strategies (4–6). Numerous studies have demonstrated that dysbiosis and variation in the gut microbiota can reduce the effectiveness of ICI (7–9). Patients with advanced melanoma treated with an anti-PD-1 mAb or an anti-CTLA-4 mAb alone or in combination with chemotherapy and treated with antibiotic (ATB) within 30 days of ICI treatment had reduced both progression-free survival (PFS) (10) and overall survival (OS) (11). In patients with non–small-cell lung cancer with ≥50% PD-L1 expression, ATB use impacted the efficacy of ICI (12). Another study examined the effect of ATB on 234 patients with several solid cancers treated with ICI and showed that ATB may affect the clinical outcomes of those patients (13).

We conducted a systematic review and meta-analysis to explore the impact of ATB use on GI cancer patients undergoing treatment with ICIs. The gut microbiota, which greatly influences the response to ICIs, can be disrupted by ATB use. Considering that the GI tract harbors the largest reservoir of gut microbiota, it is particularly vulnerable to the effects of ATBs. Our aim was to examine the specific effects of ATB use in GI cancer patients receiving ICIs.

## 2 Materials and methods

### 2.1 Registration

The study protocol was registered with the International Prospective Register of Systematic Reviews (PROSPERO) and can be accessed at https://www.crd.york.ac.uk/prospero/display_record.php?RecordID=462172.

### 2.2 Searching strategy

The study was conducted according to the (PRISMA) guidelines (14). The systematic review was performed using MEDLINE, Cochrane, Scopus, EBSCO, Web of Science. No date restrictions were applied, and all studies published till September 2023 and were published in English were included. The following terms were used (“Immune checkpoint inhibitors,” “ICIs,” “PD-1,” “ipilimumab,” “pembrolizumab,” “nivolumab,” “gastrointestinal cancers,” “esophageal cancer,” “gastric cancer,” “colorectal cancer,” “pancreatic cancer,” “liver cancer,” “gallbladder cancer,” “small intestine cancer,” “Antibiotic,” and “Protein Synthesis Inhibitors”). Studies were screened by three independent investigators and conflicts were addressed by a fourth investigator.

### 2.3 Study and patients’ criteria

Studies that are narrative reviews, systematic reviews, *in vitro* studies, editorial or animal studies were excluded from the meta-analysis. The following criteria were included: 1- Patients with GI cancer such as esophageal cancer, gastric cancer, colorectal cancer, pancreatic cancer, liver cancer, gallbladder cancer and small intestine cancer treated with ICIs therapy (PD-1, PD-L1, or CTLA-4 inhibitors) or in combination with other systematic therapies. 2- Treatment with ATBs, 60 days before or after ICI administration. Within the reviewed studies, most of the studies considered the critical window of using ATB as 30 days before or after the commencement of ICIs treatment. However, one study opted for a broader timeframe of 60 days preceding the initiation of treatment. To ensure comprehensive coverage of relevant studies, we utilized the longer 60-days window to encompass all potential research findings. 3- Control group: Patients who did not receive the intervention (ATBs). 4- Outcomes: OS and PFS were the main reported outcomes.

### 2.4 Data extraction

All of the variables and the included data were extracted and entered into Microsoft Excel spreadsheet (Microsoft, Redmond, WA, United States). The included data were reviewed and extracted by three independent investigators (IBSA, AMA, and HA) and then reviewed by two investigators (FMA and SK). The following information were extracted: Title of the study, first author, year of publication, published journals, type of the study, countries, study design, time and types of ATB, age of the patients, gender, sample size, type and stage of cancer, ICI types, outcomes like OS and PFS.

### 2.5 Quality assessment

The majority of the included studies were retrospective in nature and quality assessment was done using the Newcastle-Ottawa scale (NOS) and independently performed by two investigators (IBSA and AMA).

### 2.6 Statistical analysis

Data were analyzed using RevMan 5.3 software. Hazard Ratio (HR) and 95% Confidence Intervals were used for comparison between ATBs use and no ATBs use. Cochran’s *Q*-test and I^2^ statistics were used to assess heterogeneity between studies. If *p*-value of Cochran’s Q test is significant and I^2^ statistics higher than 50%, Random- Effect model will be applied. Otherwise, fixed effect model will be suitable. Outcome will be a forest Plot and funnel Plot for overall survival and forest Plot and funnel Plot for Precession-Free Survival (only 7 studies). Publication Bias was assessed using funnel Plot.

## 3 Results

### 3.1 Selection of eligible studies

**Figure 1:**
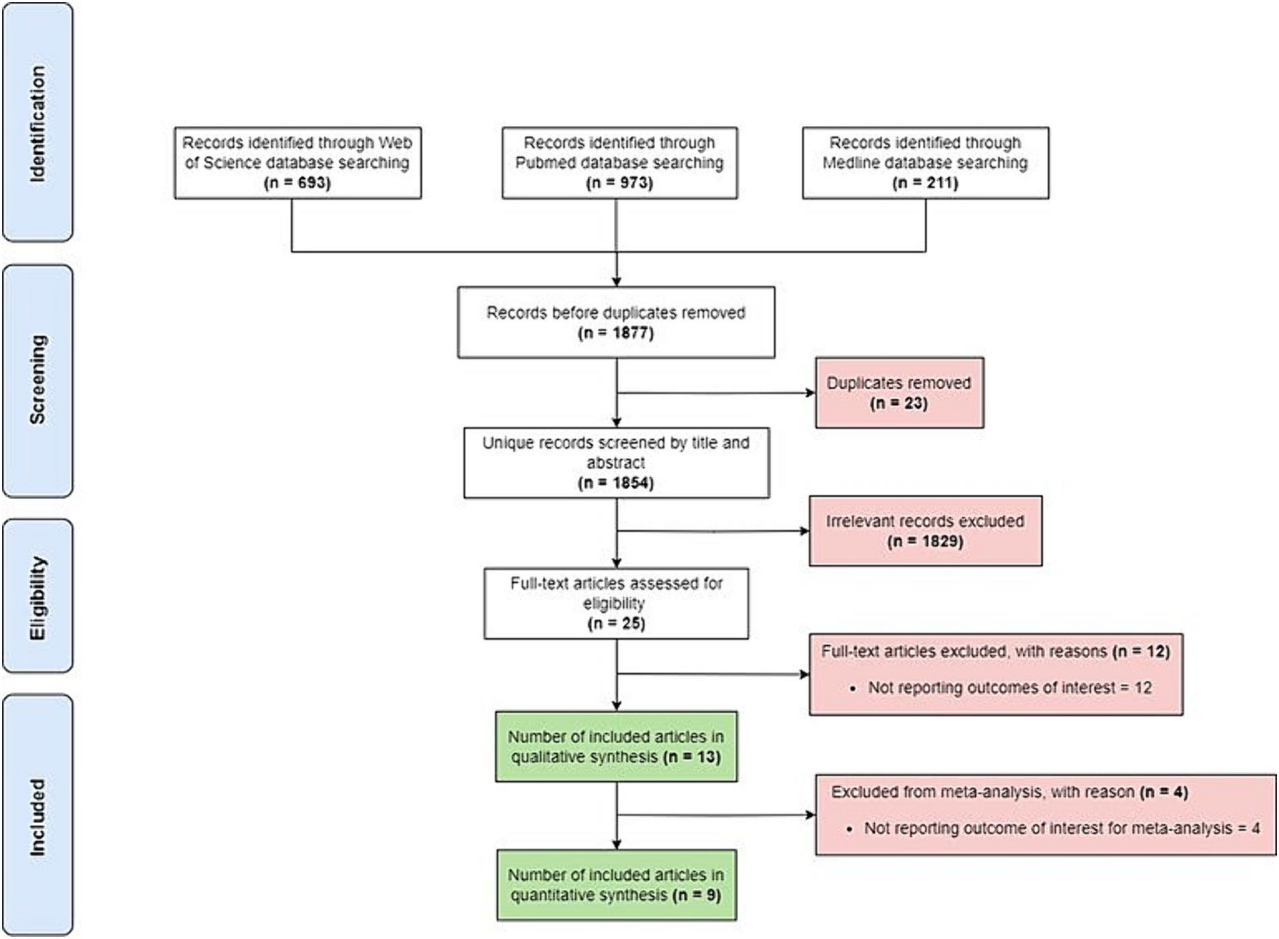
PRISM diagram of included and excluded studies.

Initial search of the included data-bases retrieved a total of 1,877 studies. There were 23 duplicates, and 1829 were excluded based on the title or abstract. Additionally, 16 studies were excluded for not reporting outcomes of interest or not reporting relevant outcome of interest for this meta-analysis. Thus, nine studies were included in the final meta-analysis (Figure 1).

### 3.2 Characteristics of studies and patients included

The nine included studies in the meta-analysis were published between 2019 and 2023 from different countries (Table 1). A total of 2,214 patients were included in the mate-analysis (Table 1). One study was an international collaboration between three European countries (Germany, Austria and Switzerland) (17). All studies were retrospective except for one which recruited patients from nine clinical trials (21) (Table 1). The meta-analysis included esophageal squamous cell carcinoma (ESCC) with 109 cases (4.9%), esophagogastric cancer (EGC) with 85 cases (3.8%), unclassified liver cancer with 610 cases (27.5%) and hepatocellular carcinoma (HCC) with 1,410 cases (63.6%) as the most common GI cancers treated with ICIs (Table 1). With an average percentage of 86.2%, men were the dominant sex, and the median age was 68.5 years. Five studies reported advanced cancer, one reported early cancer, and three did not indicate the stage of cancer. The majority of cancer cases were in this advanced stage. Of the 2,214 patients, 434 (19.6%) received ATBs treatment within 60 days prior to or following their ICI treatment. Several studies reported the use of different ATBs classes such as Aminoglycosides, Quinolones, Carbapenems, Cephalosporins, Penicillins, and Macrolides (Table 1). The majority of the studies reported the use of anti-PD(L)1 monoclonal antibodies and only one study reported using anti-CTLA-4 monoclonal antibodies (Table 1).

**Table 1:** Baseline and patients characteristics of included studies in the meta-analysis.

Study type and ID	Country and study duration	Sample size T:ATB+/ATB-	Age	Gender M/F	Cancer type	Stage of caner	ICI type	Antibiotics type
Retrospective cohort, Wang et al. (15)	China, June 2018—October 2020	215: 41/174	**≥ 65 ATB+:** 9.8% **≥ 65 ATB-:** 17.2%	87.9%/12.1%	Liver	Majority Advance	Anti-PD-1 PD-L1	Aminoglycoside Quinolones Carbapenems Cephalosporins
Retrospective cohort, Hatanaka et al. (16)	Japan, September 2020—April 2022	427: 43/384	74 (68–79)	80.5%/19.5%	HCC	Majority Advance	Anti-PD-L1	Quinolones Penicillin Cephalosporins
Retrospective cohort, Spahn et al. (17)	Germany, Austria and Switzerland August 2015—December 2019	99: 21/78	69 (20.8–87)		HCC		Nivolumab Pembrolizumab	
Retrospective cohort, Zhang et al. (18)	China, 2017–2021	85: 35/50	**≥ 65 ATB+:** 48.6% **≥ 65 ATB-:** 40%	77.6%/22.4%	Esophagogastric Cancer (EGC)	Advanced	Anti-PD-1 PD-L1	Cephalosporins Macrolides Carbapenems Quinolones
Retrospective cohort, Kim et al. (19)	Korea, 2015–2019	60: 15/45	68 (52–76)	93.3%/6.7%	Esophageal Squamous Cell Carcinoma (ESCC)	Early	Nivolumab Pembrolizumab	
Retrospective cohort, Guo et al. (20)	China and Taiwan, August 1, 2015—December 31, 2017	49: 21/28	56.7 (37.2–82.8)	98%/2%	Esophageal Squamous Cell Carcinoma (ESCC)		Anti PD-1 PD-L1	
Clinical Trails, Pinato et al. (21)	USA, 2016–2019	825: 129/696	**ATB+:** 63 (24–88) **ATB-:** 64 (18.-88)	83%/17%	HCC		Anti PD-1 PD-L1 Anti-CTLA-4	
Retrospective cohort, Cheung et al. (22)	China, January 2014—December 2019	395: 109/286	61 (52.8–69.3)	84.8%/15.2%	Liver	Advance	Nivolumab Pembrolizumab ipilimumab	Penicillin Cephalosporins Carbapenems , and others
Retrospective cohort, Alshammari et al. (23)	Saudi Arabia, 2016–2019	59: 20/39	72 (65–79)	86.4%/13.6%	HCC	Advance	Nivolumab sorafenib	

### 3.3 Impact of ATBs use on clinical outcomes in patients with GI cancer treated with ICI

**Figure 2:**
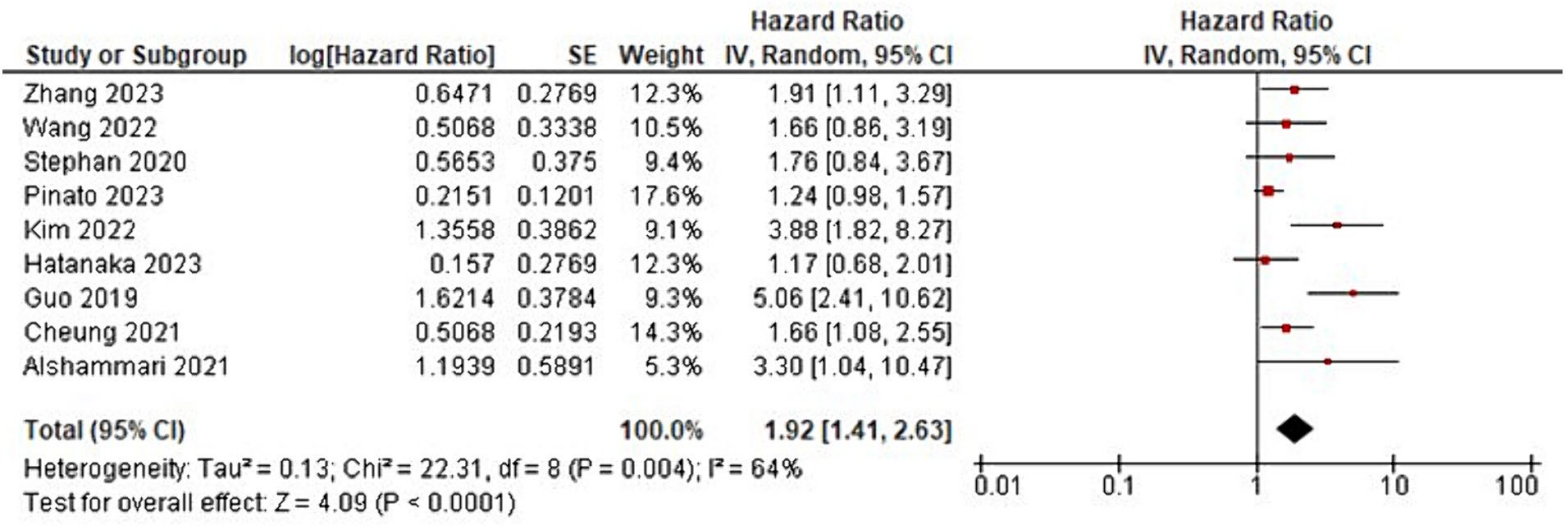
Forest plot analysis of overall survival (OS) in a comprehensive meta-analysis: evaluating treatment effects and heterogeneity.

**Figure 3:**
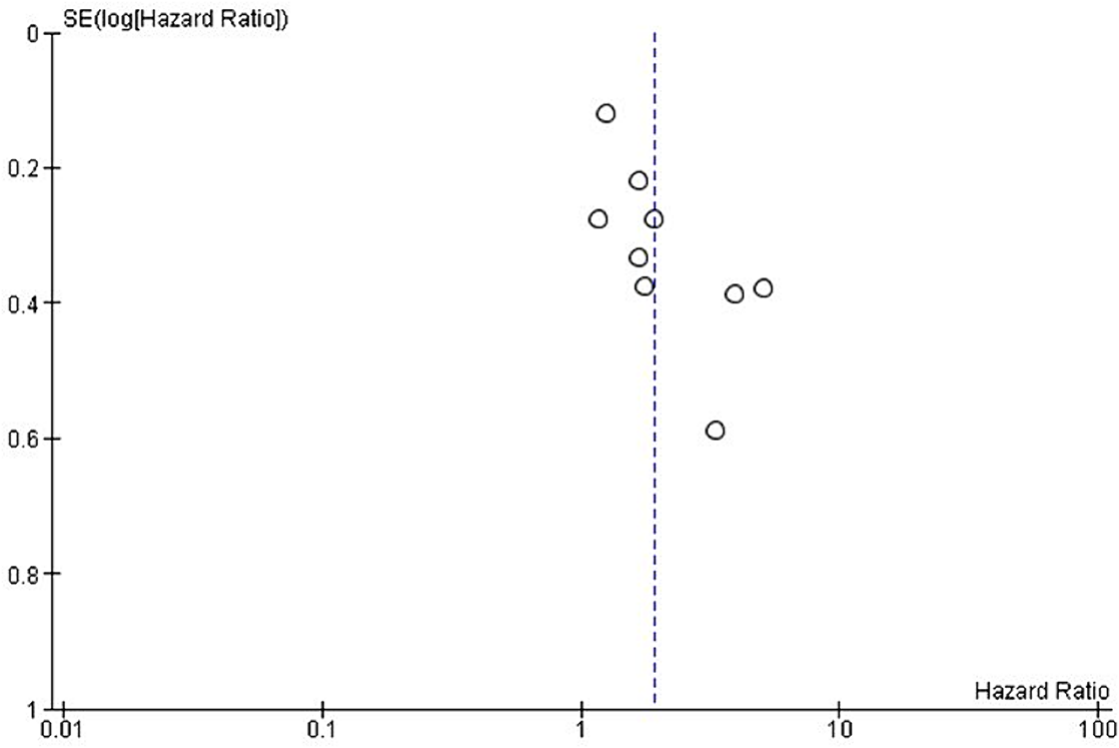
Exploring publication bias and small study effects through funnel plot analysis of overall survival in the included studies.

**Figure 4:**
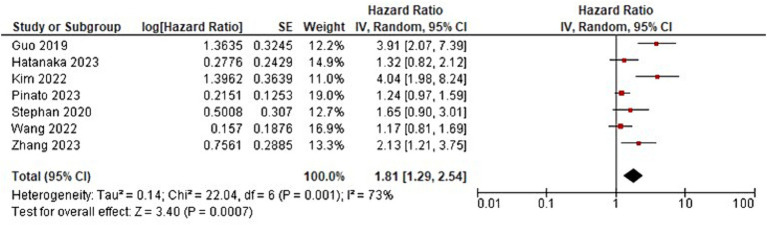
Forest plot analysis of progression-free survival (PFS) in a comprehensive meta-analysis: evaluating treatment effects and heterogeneity.

**Figure 5:**
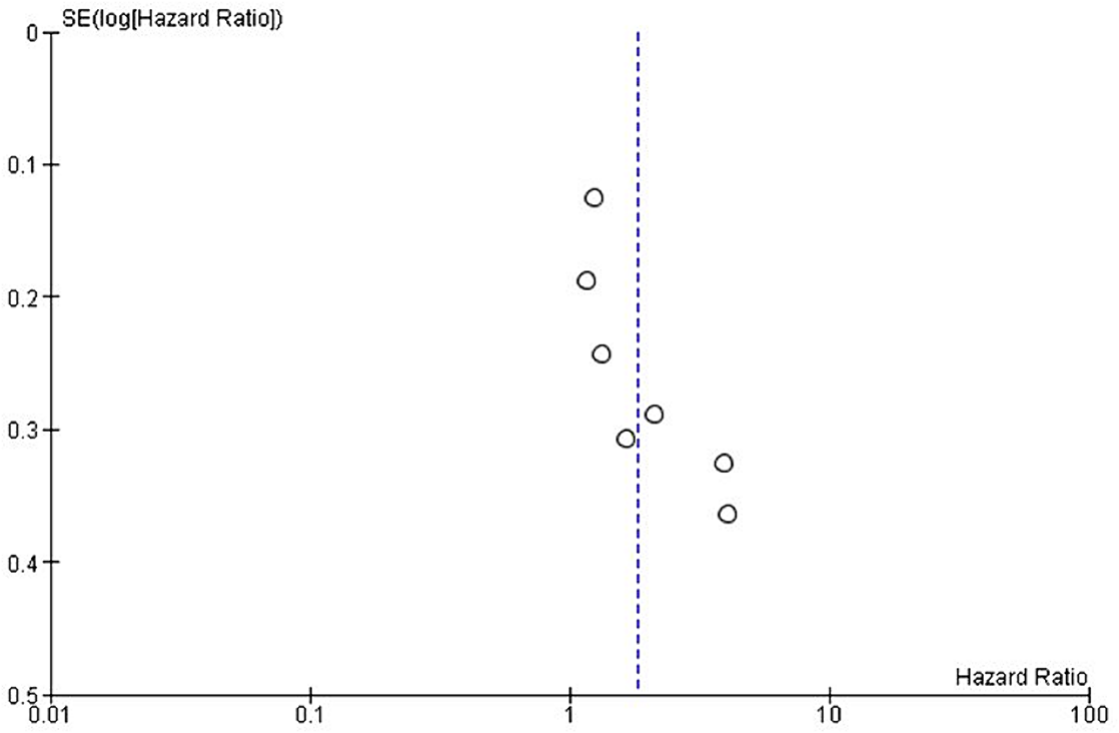
Exploring publication bias and small study effects through funnel plot analysis of progression-free survival in the included studies.

The meta-analysis showed that patients with GI cancer treated with ICI had a significantly lower overall survival rate when using ATBs than when patients did not receive ATBs (HR = 1.92; 95% CI, 1.41–2.63; *p* < 0001; Figure 2; Table 2). Significant heterogeneity was observed across the studies in terms of OS as shown by I^2^ value of 64% (*p* = 004) (Figure 3). Furthermore, ATBs use influenced negatively the PFS in GI cancer patients treated with ICI (HR = 1.81; 95% CI, 1.29–2.54; *p* < 0007; Figure 4). Significant heterogeneity was observed across the studies in terms of PFS as shown by *I*^2^-value of 73% (*p* = 001) which indicates differences in study results (Figure 5).

**Table 2:** Comparison of overall survival (OS) and progression-free survival (PFS) in studies with and without adjuvant therapy of the included studies in the meta-analysis.

Study ID	ATB exposure duration (within xx days before or after ICIs)*	OS:M 95% ATB+	OS: M 95% ATB-	PFS: M 95% ATB+	PFS: M 95% ATB-
Wang et al. (15)	30 days			5 months 95% (2.8–7.1)	5.6 months 95% (4.8–6.3)
Hatanaka et al. (16)	30 days			3.8 Months 95% (2.9–7.7)	7 months 95% (6.2–8.3)
Spahn et al. (17)	30 days	21.1 months	17.4 months	5 months	7.6 months
Zhang et al. (18)	30 days	3.04 months 95% (1.9–4.8)	4.9 months 95% (3–7.8)	2 months 95%(1.5–2.8)	3.1 months 95% (2–5.19)
Kim et al. (19)	30 days	1.7 months	8.2 months	0.8 months	2.2 months
Guo et al. (20)	60 days before and 30 days after ICIs	3 months 95% (1.5–4.5)	10.4 months 95% (8–12.8)	1.3 months 95% (1.1–1.5)	2.8 months 95% (1.1–4.5)
Pinato et al. (21)	30 days	10.7 months 95% (9.1–11.8)	11.4 months 95% (10.6–12.1)	8.3 months 95% (6.8–8.9)	8.2 months 95% (7.6–7.4)
Cheung et al. (22)	30 days	Not reported in months			
Alshammari et al. (23)	30 days	5 months 95%(3.2–6.7)	10 months 95% (0–22.2)		

*Within 30 days before or after ICIs unless indicated otherwise.

### 3.4 Bias assessment of the included studies

Publication bias was assessed using funnel plots. The selected studies were evenly scattared around both sides in both OS and PFS and there was no significant publication bias for OS (Figure 3) and PFS (Figure 5).

## 4 Discussion

An expanding body of evidence indicates a significant association between the gut microbiota and immune response. The use of ATBs is known to disrupt the balance of microbial communities, leading to a condition called dysbiosis. Dysbiosis is characterized by a decrease in microbial diversity and the depletion of beneficial bacterial species that play crucial roles in activating and maintaining the immune system (24). These alternations can result in a compromised host immune system and potentially diminish the therapeutic effectiveness of ICIs (8). Additionally, a specific study has demonstrated that ATB-induced dysbiosis can lead to a decline in intestinal immune cell functions (25). These intricate interactions underscore the importance of gaining a better understanding of how ATB interventions during ICI treatment may impact overall patient response.

The results of this meta-analysis provided insight into the effects of ATBs use on treatment outcomes on patients treated with immunotherapy for GI cancer. The incorporation of research from various nations expands the viewpoint and enhances the applicability of the findings. The meta-analysis’s preponderance of HCC cases highlights the pressing need for efficient treatment options for this difficult-to-treat illness (26, 27). The high percentage of patients with advanced-stage cancer emphasizes how crucial it is to investigate cutting-edge treatment options, like ICIs, in order to enhance patient outcomes.

The fact that 19.6% of the patients were prescribed ATBs and the negative impact of this intervention raises concerns regarding the possible impact of these drugs on the effectiveness of immunotherapy (28, 29). It has been demonstrated that the gut microbiota, which is essential for regulating immune responses, is impacted by ATBs (30). Immunotherapy may be less effective if the composition of the microbiota is disturbed because this can hinder the immune system’s capacity to mount an effective anti-tumor response.

The meta-analysis’s findings show that when ICI were used to treat GI cancer, the use of ATBs significantly affects clinical outcomes (Figures 2, 4; Table 2). The results imply that in this patient population, the use of ATBs is linked to lower overall and progression-free survival. Patients with GI cancer treated with ICI who received ATBs had a significantly lower overall survival rate (HR = 1.92; 95% CI, 1.41–2.63; *p* < 0.001). These findings are in line with earlier research that found comparable links between the use of ATBs and a lower overall survival rate in cancer patients (31, 32). Changes in the gut microbiota and their subsequent effects on immune responses may be the reason why ATBs have a negative effect on survival outcomes (33).

The I2 value of 64% (*p* = 0.004) suggests that there is heterogeneity among the studies. This is a common challenge in meta-analyses and can be caused by variations in study design, patient populations, treatment protocols, and other factors (34). It is imperative to take into account these sources of heterogeneity when interpreting the findings of the meta-analysis. It might be possible to pinpoint particular elements that contribute to the observed differences in results with more investigation into the underlying causes of heterogeneity.

Additionally, the meta-analysis showed that the use of ATBs had a negative impact on PFS in patients with GI cancer receiving ICI (HR = 1.81; 95% CI, 1.29–2.54; *p* < 0.007). These results are consistent with earlier studies showing that exposure to ATBs may impair the efficacy of ICI therapy in treatment of advanced cancers (35–37). This detrimental effect could be exacerbated by immune dysregulation brought on by disruption of the gut microbiota (38). However, heterogeneity was found among the studies evaluating PFS, with an *I*^2^-value of 73% (*p* = 0.001), similar to the overall survival analysis. This high level of heterogeneity raises the possibility that different study designs or patient characteristics could have had an impact on the results (34). Additional exploration of the heterogeneity’s sources can shed light on the variables influencing the variation in PFS outcomes.

This meta-analysis is limited as most of the included studies were retrospective in nature and thus this may introduce potential confounders and inherent biases (39). To offer more conclusive proof about the effect of antibiotic use on immunotherapy outcomes in patients with GI cancer, prospective studies and randomized controlled trials are necessary.

A range of ATBs were used including aminoglycosides, quinolones, carbapenems, cephalosporins, penicillin, and macrolides (Table 1). Due to the small number of studies, this meta-analysis lacked comprehensive information regarding the prevalence of or use of specific ATBs within each class. It is therefore challenging to ascertain which ATBs were most commonly used or to make direct comparisons between them.

The meta-analysis’s findings raise important concerns regarding the use of ATBs in GI cancer patients receiving ICI therapy. When prescribing antibiotics to this patient population, caution should be exercised due to the potential negative effects on both PFS and OS. It is crucial to realize that, despite the meta-analysis’s finding of an association, there is no proof that the use of ATBs and clinical outcomes are causally related. Numerous factors could account for the observed associations. ATBs may alter the gut microbiota, which is crucial for controlling immune responses and may have an impact on the effectiveness of immunotherapy (4). Furthermore, underlying infections or comorbidities that may negatively impact treatment outcomes may be reflected in the ATBs indication.

To clarify the underlying mechanisms and investigate potential confounders that might contribute to the observed associations, more research is required. To validate these results, take into consideration possible confounding variables, and provide more insight into the impact of ATBs on the effectiveness of ICI therapy in patients with GI cancer, prospective studies and randomized controlled trials are necessary.

It is important to note that the GI tumor itself can influence the composition of the gut microbiota, thereby impacting the response to ICIs treatment. Several studies suggest that certain GI tumors, like CRC, can induce changes in the gut microbiota through interactions within the tumor microenvironment. For instance, a study investigated fecal and mucosal samples from 59 patients undergoing surgery and compared them to healthy controls, revealing differential associations between CRC-associated microbiota and the expression of host immunoinflammatory response genes (40). These findings suggest that the tumor’s immune environment and response to ICIs could potentially be influenced by tumor-induced changes in the gut microbiota (40). Similarly, other studies have characterized tumor-associated microbiota in HCC patients and have identified potential contributions of microbiota and gut epithelial barrier functions to HCC pathology (41–43). These observations highlight the potential role of tumor-induced microbiota changes in determining the response to ICIs. Further research should investigate the direct effects of GI tumors on the microbiota and its consequent impact on ICI response.

Furthermore, future studies should examine the role of ATB use in specific subgroups of cancer patients, such as those with microsatellite instability high (MSI-H)/deficient mismatch repair (dMMR) cancer. This subpopulation has shown a significant response rate to ICIs, but not all MSI-H/dMMR patients exhibit a favorable response. Therefore, further investigation is needed to enhance the response rate in this subgroup. A single study indicated no correlation between lower response rates or overall survival in MSI-H/dMMR patients with metastatic CRC who received ATBs around the initiation of ICIs treatment. However, this finding requires confirmation in a larger prospective cohort (44).

In summary, this meta-analysis’s findings pointed to a negative correlation between the use of ATBs and clinical outcomes in GI cancer patients receiving ICI, such as overall survival and progression-free survival. These results highlight the necessity of using caution when prescribing ATBs to this patient population and the significance of further research to elucidate the underlying mechanisms and improve treatment approaches for patients with GI cancer undergoing immunotherapy.

## Data availability statement

The original contributions presented in the study are included in the article/supplementary material, further inquiries can be directed to the corresponding authors.

## Author contributions

FA: Conceptualization, Data curation, Formal analysis, Funding acquisition, Investigation, Methodology, Project administration, Resources, Software, Supervision, Validation, Visualization, Writing – original draft, Writing – review & editing. IA: Writing – review & editing, Visualization, Validation, Software, Resources, Project administration, Methodology, Data curation. AA: Writing – review & editing, Validation, Software, Methodology, Data curation. HA: Investigation, Methodology, Software, Validation, Writing – review & editing. SK: Data curation, Formal analysis, Methodology, Software, Validation, Writing – review & editing. KA: Conceptualization, Investigation, Validation, Visualization, Writing – review & editing. JA-T: Conceptualization, Formal analysis, Investigation, Methodology, Resources, Software, Supervision, Validation, Visualization, Writing – original draft, Writing – review & editing.
